# Structural behaviour of built-up I-shaped CFS columns

**DOI:** 10.1038/s41598-024-77455-x

**Published:** 2024-10-27

**Authors:** Ardalan B. Hussein

**Affiliations:** Department of Structural Engineering and Geotechnics, Széchenyi István University, Egyetem Tér 1, Győr, 9026 Hungary

**Keywords:** Axial strength, Back-to-back C-sections, Built-up I-sections, Buckling, Cold-formed steel (CFS), Critical elastic buckling load, Screw spacing, Fastener spacing, Lipped channels, Finite element analysis (FEA), Direct strength method (DSM), Engineering, Civil engineering

## Abstract

The utilization of cold-formed thin-walled members as structural members has gained significant popularity due to their advantages in fabrication, cost-effectiveness, and transportation convenience. However, the reduced thickness of the used sections poses challenges such as global, local, and distortional member buckling, leading to a decrease in their axial strength. This study focuses on addressing these challenges by connecting the channels together using screws as an alternative to welding, considering the cost, time, and ease of implementation. Conducting finite element analysis on structural columns built-up from cold-formed double C steel channels and subjected to axial loads, this paper verifies the numerical models used against experimental tests known from the literature. A comparison of experimental results with nonlinear FEA and AISI & AS/NZ standards reveals commendable agreement, particularly in predicting the buckling behavior of the built-up I-shaped CFS columns. While the results of the finite element analysis show an overestimation of approximately 3.6% compared to the experimental tests, the AISI and AS/NZS standards demonstrate a conservatism of about 3.0%. Furthermore, the current study investigates the influence of screw spacing on axial strength of built-up cold-formed steel columns. The findings are derived from 175 finite element experiments, evaluating seven different cross-sectional profiles with twelve distinct screw spacings. These spacings correspond to the half-wavelength of local, distortional, and global buckling, divided by values ranging from one to four. The screw spacing determined by half the local buckling half-wavelength along the webs’ centerline resulted in enhancements of 22%, 7%, 13%, and 11% in the critical elastic local, distortional, and global column buckling loads, as well as the nominal axial strength, respectively. These increases were even more pronounced for double-lane fasteners with the same spacing, yielding improvements of 25%, 46%, 17%, and 12%, respectively. For economic considerations, it is advisable to utilize single-lane fasteners with a half-wavelength equal to half the local buckling half-wavelength.

**Supplementary Information:**

The online version contains supplementary material available at 10.1038/s41598-024-77455-x.

## Introduction

Cold-formed steel is becoming increasingly popular in construction due to its low cost^1^, light weight^2^, high strength-to-weight ratio^3,4^, and ease of manufacturing. The built-up I-shaped CFS column is an example of a cold-formed steel component used in trusses^5,6^, residential and commercial construction. By examining different C-section configurations and varying screw spacings, the study determines the optimal screw spacing, resulting in improved strength characteristics. Using the Abaqus software, the effects of screw quantity on the structural behavior of a I-shaped CFS column will be investigated. The results of this study will be beneficial for designing cold-formed steel structures and will allow engineers to make more informed design choices.

Screws that self-taper and self-drill are the two most common varieties. For better load bearing capability or to make the fastening self-sealing, most screws will be used in combination with washers. Certain varieties feature plastic heads or plastic caps, which are available for added corrosion protection and/or color matching^7^. By drawing the screw out of the plate (pull-out), dragging material over the screw head and washer (pull-over), or by tension-fracturing the screw^8^, demonstrated how screw connections exposed to tension might fail^9^. Illustrated how fastener efficacy is substantially influenced by the end support conditions of built-up sections. The spacing, placement, and stiffness of fasteners, as well as changes in the bending moment and axial force diagrams, all affect the effective flexural rigidity, which is not a constant throughout the length of the beam. The^10^ states that while the distance between screw centers must be at least three times the nominal screw diameter (d_f_), it must also allow room for screw washers. A screw must have a minimum distance from the center to the edge of any section of 1.5 d_f_. So, 3.0 mm ≤ d_f_ ≤ 7.0 mm. Nevertheless^11^, suggests that 2.03 mm ≤ d_f_ ≤ 6.35 mm. Moreover, in accordance with^12^, screw fasteners should be deemed to be 80% effective in steel-to-steel connections when the center-to-center spacing is less than 3 times the nominal diameter but larger than or equal to 2 times the nominal diameter.

In the case of compression members with two sections in contact^13^, a modified slenderness approach may be used:

1 $$\:{\left(\frac{\text{K}\text{L}}{\text{r}}\right)}_{\text{m}}=\sqrt{{\left(\frac{\text{K}\text{L}}{\text{r}}\right)}_{\text{o}}^{2}+{\left(\frac{\text{a}}{{\mathbf{r}}_{\text{i}}}\right)}^{2}}$$

where r_i_ is the minimum radius of gyration of the whole unreduced cross-sectional area of a certain form in a built-up member, a is the intermediate fastener or spot weld spacing, K is the effective length factor, laterally unbraced length of member, radius of gyration of full unreduced cross-section about axis of buckling, and $\:{\left(\frac{\text{K}\text{L}}{\text{r}}\right)}_{\text{o}}$ is the overall slenderness ratio of the entire section about the built-up member axis. According to research^14^, the modified Direct Strength Method (DSM) offers more accurate predictions than the present DSM. The study’s validation was completed by^15^. Experimental values were discovered to be 1.072 times the FEM values from the validation research, and DSM values to be 0.97 times the FEM values. The suggested design guideline and the modified DSM value are appropriate for designing back-to-back stiffened columns based on the important study findings.

According to the findings of research^16^, the direct strength method’s real global buckling equation is already adequate to account for the distortional-global (DG) buckling interaction in the case of lipped channel columns^4^. demonstrated that single-channel sections failed due to local buckling, which started from the flanges towards the web, as well as flexural buckling after the yielding limit was reached. The axial capacity obtained from the tests and FEA also demonstrated that design in accordance with the (DSM) is accurate and conservative by only 4% on average^17^. Claimed that the plate buckling at the mid-height causes the doubly symmetric plain section bolted back-to-back to fail. As^18^ predicted, lipped channels utilize the material more effectively than plain channels over the whole range of lengths, thicknesses, and load eccentricities.

The objective of this research is to analyze the influence of screw spacing and configuration to provide valuable insights for optimizing the axial load capacity of built-up cold-formed steel (CFS) C-section columns. The findings indicate that both the quantity and spacing of screws play a significant role in enhancing the strength of built-up I-shaped CFS columns, particularly with regard to their buckling resistance.

## Special behaviour of built-up I-shaped CFS columns

The finite element (FE) and finite strip (FS) methods are the predominant numerical techniques used for analyzing the buckling behavior of CFS structural members. Two separate kinds of CFS member behaviors have been investigated throughout the finite element method. Initially, the linear elastic buckling analysis is used to calculate the crucial elastic buckling loads. Furthermore, a nonlinear elastic buckling analysis is performed in order to accurately capture the post-buckling behavior^19^. An analysis of the buckling characteristics of built-up I-shaped CFS columns subjected to compressive loads indicates three possible modes of failure: global (G), which includes flexural, torsional, or flexural-torsional buckling; local (L) buckling; and distortional (D) buckling. Alternatively, failure might arise from the combined effect of all three types^20^.

Plate buckling at mid-height is a potential failure mode for built-up I-shaped CFS columns^17^. Regardless of the space between connectors, it has been discovered that contact pressure between components has only a little impact on the final capacity, despite other factors being taken into account^21^. The average values for dynamic coefficients of friction for combinations in air, such as “coated steel,” vary between 0.14 and 0.42. These values are influenced by the amount of contact pressure^22^. Furthermore, both experimental testing and finite element analysis have shown that thin fixed-ended beam-column members usually are not affected by torsional or flexural-torsional buckling modes^23^. In conclusion, within the scope of this paper, the analysis conducted on the 175 Finite Element Method (FEM) models reveals comprehensive consideration of both normal and tangential behaviors in the contact property section.

### Effect of slenderness ratio

The modified slenderness ratio given by Eq. (1) is useful mainly in areas with minimal fastener spacing, as Mahar et al.^24^ found. However, when fastener spacing grows, their investigation shows that it becomes too cautious. On the other hand, results from^25^ demonstrate that for built-up back-to-back CFS columns with short screw spacing, the modified slenderness approach, when applied to global buckling, results in dangerous ultimate strength predictions.

Furthermore, as shown by^26^, the local buckling strength of built-up I-shaped CFS columns is about double that of the equivalent C-section components. This conclusion is consistent with the evidence presented in^27^, which shows that the ultimate strength and stiffness of built-up I-shaped members that fail as a result of the interplay between local, distortional, and overall buckling are highly influenced by the slenderness ratio. Additionally, a few effects on the assembled I-shaped members break down in the presence of the local-distortional buckling interaction.

### Correlation between end fastener groups and buckling resistance

End fastener groups (EFGs) must be positioned longitudinally at a maximum spacing of four times the diameter of applied screws and over a distance equivalent to 1.5 times the maximum dimension of the built-up Sects^13,28^. Nevertheless, studies have shown that EFG could be too cautious and redundant in cases where the end supports are either fixed or pinned, and warping is already addressed^29^. When the load is applied using inflexible end platens, both sets of fasteners and welds at the ends show little impact^30^. In addition, the spacing between screws and the presence of EFG have little effect on the compression behavior and capacity of NC members, as stated in reference^31^.

Furthermore, the use of EFGs may boost capacity by up to 33%. However, it may also elevate member reliability indices in comparison to columns that lack EFGs. However, when local buckling and global buckling interact, both the capacity for buckling and the deformations caused by bending suffer a slight decrease, and the effectiveness of the EFG is also reduced^32^. The impact of the EFG becomes more prominent when shear slip decreases, resulting in an elevation of composite action^28^.

Although EFGs and other configurations of built-up back-to-back CFS section connecting by fasteners (built-up stud fasteners) may greatly increase the overall resistance to buckling^28,33^, it is important to recognize that these layouts and the degree of composite action they provide do not improve the capacities for local and distortional buckling^9,33,20^.

### Accuracy of AS/NZS and AISI-S100 standard formulas

For various column kinds, the AISI^11,13^ and AS/NZ^10^ Standards show differing degrees of conservatism in their projections. In particular, these criteria are shown to be non-conservative for stub columns^34,35^, since they failed due to local buckling. Their conservatism, on the other hand, is clearly seen in short, intermediate, and slender columns when failure is caused by a combination of global and local buckling.

In contrast, the AISI and AS/NZS standards exhibit a distinct conservative trend, as shown in^36^. By around 12%, they are considered non-conservative when stubs and short columns fail because of local buckling. For the strength of intermediate and slender columns, these criteria, however, turn out to be too cautious, failing mostly due to global member buckling^36^.

Additional findings, as described in^35^, show that the AISI and AS/NZS standards over-conserve about 15% of the time when built-up columns collapse due to global buckling. On the other hand, these guidelines are around 8% less cautious for built-up columns that collapse mainly because of local buckling. When examining face-to-face built-up columns, the degree of conservatism in the criteria differs according to the failure mechanism. They are about 15% more cautious for global buckling and about 5% less conservative for local buckling^37^.

When the Direct Strength Method’s modified strength formula is applied, it is found that this method accurately predicts the ultimate strength of intermediate slender columns, which experience a combination of local and distortional buckling before failing in the distortional mode^38^.

It is determined that this technique offers good forecasts of the failure load for CFS lipped channel columns experiencing local-distortional-global buckling interaction^39,40^, while evaluating the efficacy of the presently codified DSM design curve against local-global interactive buckling.

When completely composite buckling behavior is guaranteed, the modified AISI slenderness formula, see Eq. (1), is shown to be conservative by around 10% when looking at the buckling load prediction for built-up Sect^41^.

In the end, it is shown that for columns with intermediate fastener spacing less than the appropriate local buckling half wavelength, the design forecasts are ultimately conservative. For columns with wide connectivity spacing, on the other hand, they are non-conservative^42^.

### Effect of screw spacing and arrangement

The failure mechanisms of steel-to-steel screw connections include screw hole bearing failure, screw shear failure and screw pull-out from steel plates. There is a link between the failure modes and the thicknesses of steel plates and the diameters of screws^43^. Furthermore, the placement and configuration of fasteners are significant factors in ascertaining the failure load of members. Nevertheless, accurate calculations using current experimental data continue to pose a challenge^44^. It is worth mentioning that the ultimate strength of built-up I-shaped CFS columns can be significantly influenced by the spacing of screws and the configuration of the end fastener group. This effect is particularly pronounced in columns that have encountered global buckling mode^27^.

According to the specified guidelines^8,10–13,45^, it is necessary for the center-to-center spacing between fasteners (such as screws and bolts) to be a minimum of three times the diameter of the fasteners. Additionally, the distance between the first and last fasteners from the initial and terminal edges of the structural members, as well as the separation between fasteners from the junction of the web and flanges, should not be less than 1.5 times the diameter of the fasteners. Conversely, to mitigate the risk of end bearing failure, it is advised in the recommendations^46^ that the distance between the first and last screws and the edges be maintained at a minimum of 20 mm.

The findings emphasize that decreasing the ratio of fastener spacing greatly improves the ability of built-up sections to resist buckling. This phenomenon is especially noticeable in both the global and local buckling areas of constructed open Sect^47^. Furthermore, it is clear that the buckling behavior of back-to-back aluminum alloy channel section columns is affected by the distance between the screws. Specifically, a smaller screw spacing results in the channels staying intact until failure^48^.

Moreover, the research^49^ shows that the spacing of screws in between has a significant influence, with its effect depending on the failure mode of CFS-built-up cross-sections. Overall, the test findings demonstrate a significant impact of the spacing between intermediate screws on both the maximum load capacity and the manner in which double C-section CFS-built-up columns fail. More precisely, a rise in the ratio of local slenderness ($\:{\varvec{\uplambda\:}}_{\text{l\:}}$) and the distance between screws (a) is linked to an increased probability of local-global interaction buckling^49^.

Moreover, the rotational rigidity of the connection between the flange and the web is improved when the distance between the webs that link the fasteners is reduced. It is important to mention that the maximum strength of sections with wide interior webs in relation to their thickness drops dramatically when the space between connecting fasteners increases. However, sections with narrow webs in relation to their thickness only exhibit a little sensitivity to fastener spacing^50^.

The impact of screw spacing varies depending on the column slenderness. When it comes to stub columns, adding more screws has a little impact on strength. However, for short and intermediate slender columns, the strength is greatly influenced by the number of screws. Specifically, when the screw spacing is doubled, the strength of short columns is reduced by around 5–10%, while intermediate slender columns see a decrease of 10–15%. Nevertheless, thin columns do not exhibit any notable disparity in strength since their failure is mostly caused by global buckling^34^.

Additionally, the axial strength of columns shows an average increase of 10% for plain parts, when the number of screws is increased from 2 to 5 ^48^. By connecting webs in back-to-back CFS members using fasteners, it is possible to achieve composite action, resulting in a capacity increase of up to 21% ^51^. This composite action mostly happens when there is solitary global buckling in the flexural behavior^32^.

The study also examines the effect of spring spacing, finding that it does not affect local buckling, has a small impact on distortional buckling, and has a significant influence on global buckling when the spring spacing is equal to or less than four times the length of critical global buckling^52^. As stated in reference^41^, in order to achieve complete composite action, the spacing between fasteners should be less than or equal to the length of the member divided by five. However, reference^19^ implies that a fastener spacing of s = L/2 is enough to improve the overall buckling load capacity, where L is the built-up column length, coming close to attaining complete composite levels.

It is crucial to acknowledge that when the distance between screws exceeds the local buckling half-wavelength of the corresponding C-section portions, it is not possible to prevent local buckling in cold-formed I-section columns^26,21,42^. However, studies conducted by to^37,29,20^ argue that the distance between screws has a little impact on the axial strength of cross-sections.

## Design criteria based on AISI and AS/NZ standards

Numerous authoritative sources provide methodologies for determining the nominal buckling load and moment of thin-walled sections, including standards such as those established by AISI S100-16 ^11,13^, AISI S240 ^12^, AISI D100-08 ^45^, AS/NZS 4600 ^10^, Eurocode 3 Part 1–3 ^7^, and the book titled Cold-Formed Steel Design^8^, written by Wei-Wen Yu et al.

The following equations are utilized to determine the buckling resistance of cold-formed steel (CFS) columns^11,13^:

2 $$\:{\text{P}}_{\text{ne\:}}={\text{A}}_{\text{g}}{\text{F}}_{\text{n}}$$

where

3 $$\:{\text{F}}_{\text{cre\:}}=\:{\text{P}}_{\text{cre\:}}/\:{\text{A}}_{\text{g}}$$

4 $$\:{{\uplambda\:}}_{\text{c}}=\sqrt{\frac{{\text{F}}_{\text{y}}}{{\text{F}}_{\text{cre\:}}}}$$

5 $$\:\:{\text{\:}\text{F}}_{\text{n}}=\left({0.658}^{{{\uplambda\:}}_{\text{c}}^{2}}\right){\text{F}}_{\text{y}}\text{f}\text{o}\text{r}\:{{\uplambda\:}}_{\text{c}}\le\:1.5$$

6 $$\:\:{\text{F}}_{\text{n}}=\left(\frac{0.877}{{{\uplambda\:}}_{\text{c}}^{2}}\right){\text{F}}_{\text{y}}\text{f}\text{o}\text{r}\:{{\uplambda\:}}_{\text{c}}>1.5$$

and where F_cre_ is the elastic global (flexural, torsional, or flexural-torsional) buckling stress, P_cre_ is the elastic global column buckling force, A_g_ is the gross area, F_y_ is the yield stress and F_n_ is the compressive strength.

For the local buckling resistance of cold-formed steel (CFS) columns has to be calculated in the following way^11,13^:

7 $$\:{\text{P}}_{\text{n}\mathcal{l}}={\text{P}}_{\text{n}\text{e}}\:\:\:\:\:\text{f}\text{o}\text{r}\:{{\uplambda\:}}_{\mathcal{l}}\le\:0.776\:\:\:$$

8 $$\:{\text{P}}_{\text{n}\mathcal{l}}=\left[1-0.15{\left(\frac{{\text{P}}_{\text{c}\text{r}\mathcal{l}}}{{\text{P}}_{\text{n}\text{e}}}\right)}^{0.4}\right]{\left(\frac{{\text{P}}_{\text{c}\text{r}\mathcal{l}}}{{\text{P}}_{\text{n}\text{e}}}\right)}^{0.4}{\text{P}}_{\text{n}\text{e}}\:\:\:\:\:\:\text{f}\text{o}\text{r}\:{{\uplambda\:}}_{\mathcal{l}}\le\:0.776$$

where

9 $$\:{{\uplambda\:}}_{\mathcal{l}}=\sqrt{{\text{P}}_{\text{n}\text{e}}/{\text{P}}_{\text{c}\text{r}\mathcal{l}}}$$

and where P_crl_ is the elastic local buckling force.

The following equations are employed to determine the distortional buckling resistance of cold-formed steel (CFS) columns^11,13^:

10 $$\:{\:\text{P}}_{\text{n}\text{d}}=\left[1-0.25{\left(\frac{{\text{P}}_{\text{c}\text{r}\text{d}}}{{\text{P}}_{\text{y}}}\right)}^{0.6}\right]{\left(\frac{{\text{P}}_{\text{c}\text{r}\text{d}}}{{\text{P}}_{\text{y}}}\right)}^{0.6}{\text{P}}_{\text{y}}\text{f}\text{o}\text{r}\:{{\uplambda\:}}_{\text{d}}>0.561$$

11 $$\:{\text{P}}_{\text{n}\text{d}}={\text{P}}_{\text{y}}\:\text{f}\text{o}\text{r}\:{{\uplambda\:}}_{\text{d}}\le\:0.561\:\:\:$$

where

12 $$\:{{\uplambda\:}}_{\text{d}}=\sqrt{{\text{P}}_{\text{y}}/{\text{P}}_{\text{c}\text{r}\text{d}}}$$

and where P_y_ is the column axial yield strength, P_crd_ is the elastic distortional column buckling force.

Finally, the nominal column resistance is^11,13^:

13 $${\text{P}}_{{{\text{DSM}}}} = {\text{ P}}_{{\text{n}}} = {\text{ min }}\left( {{\text{P}}_{{{\text{ne}}}} ,{\text{P}}_{{{\text{n}}\ell }} ,{\text{ and }}\,{\text{P}}_{{{\text{nd}}}} } \right),$$

and the design column resistance is $\:{{\upvarphi\:}}_{\text{C}}{\text{P}}_{\text{n}}$ where $\:{{\upvarphi\:}}_{\text{c}}=0.85\:$.

## Validation of finite element analysis (FEA), AISI, and AS/NZS standards

In the process of validating Finite Element Analysis (FEA) through experimental tests, this study employed two identical C-section columns, connected back-to-back to form an I-section column using self-drilling screws. To secure the columns, 15 mm thick steel end plates were welded to both ends of the test members^26^. In contrast, test members’ end plates were affixed to specially designed devices to simulate pin-pin ends. Shell elements represented all columns, while analytical rigid elements were employed for the base plates. The simply supported boundary conditions at the end supports were consistently applied across all Finite Element (FE) models of back-to-back channel (BC) members.

**Fig. 1:**
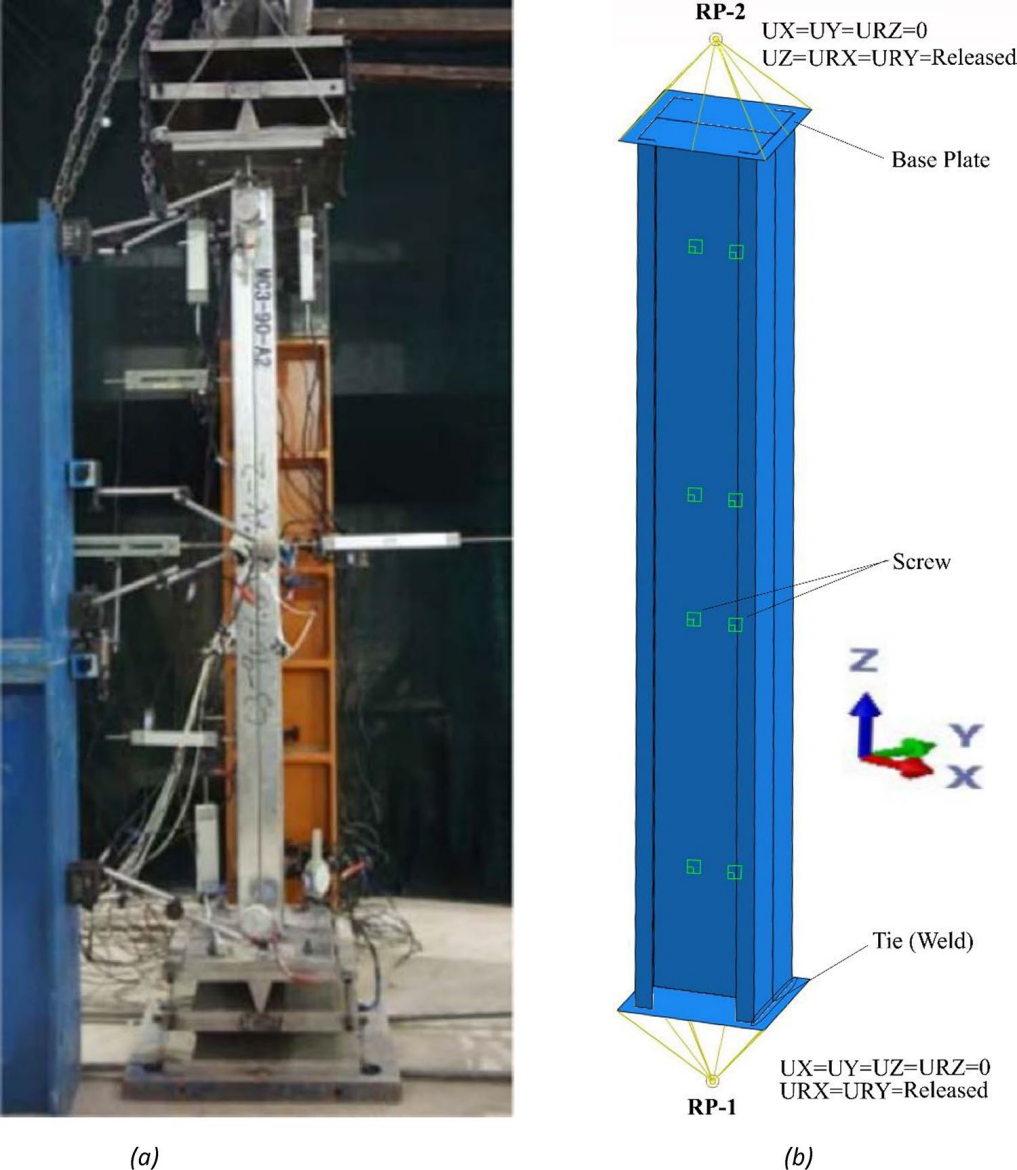
Comparison between experimental^26^ and finite element modeling (FEM) of back-to-back built-up columns.

Initially, TIE (weld) constraints were applied to each end cross-section, connecting them to the respective base plates. Subsequently, all displacements (Ux, Uy, and Uz) and rotation (URz) of the bottom reference point (RP-1) were constrained, as illustrated in Fig. 1. The top reference point (RP-2) underwent a similar treatment, except for the axial displacement (Uz), which was released. Displacement control was implemented to apply axial compression loading via RP-2. Both reference points were strategically located at the center of gravity (c.g.) of the members. Determination of (c.g.) involved meticulous calculations using AutoCAD, as opposed to conventional methods such as ETABS, SAP200, or Revit, which are commonly employed for this purpose. It is noteworthy that AutoCAD was specifically chosen for its precision in establishing (c.g.).

### Labeling scheme for test specimens

The test specimens were systematically labeled following a standardized convention to denote their section type, cross-sectional dimensions, and trial number. The adopted labeling format for the specimens was represented as “wCx-y-Az,” where each character conveys specific information about the specimen.

- “w” indicates the length category: “L” represents long lengths, “M” represents intermediate lengths, and “S” represents short lengths.
- “C” denotes the column configuration.
- “x” specifies the section type: “1” refers to single-channel sections, and “3” refers to back-to-back built-up channel sections.
- “y” represents the cross-sectional dimension, with “90” corresponding to a web depth of approximately 90 mm, and “140” indicating a web depth of approximately 140 mm.
- “Az” signifies the trial number, with “A1” denoting the first trial, “A2” the second trial, and “A3” the third trial.

For instance, the label “LC3-90-A1” represents a long back-to-back built-up channel section with a web depth of approximately 90 mm, used in the first trial.

### Mesh size and element type of validated models

When the section thickness is less than one-tenth of an elemental dimension, it is advisable to model the section as shell elements^53^. The S4R element, known for its computational efficiency, proves suitable for a diverse array of applications, especially in the analysis of thin-walled structures. In this study, a mesh size of 5 mm × 5 mm was determined through a careful convergence study.

Additionally, a “surface-to-surface” contact approach was employed to accurately simulate the interaction between individual C-sections. This strategy guaranteed a precise depiction of the physical interactions taking place throughout the loading process.

### Material properties

The cold-formed column under investigation, with thicknesses of 1.2 mm (LC3-90) and 1.5 mm (LC3-140), is composed of Chinese Q235 steel. Material properties, including yield and ultimate strengths and elastic modulus, were derived from standard tensile coupon specimens extracted along the longitudinal direction of the web of the tested sections.

**Fig. 2:**
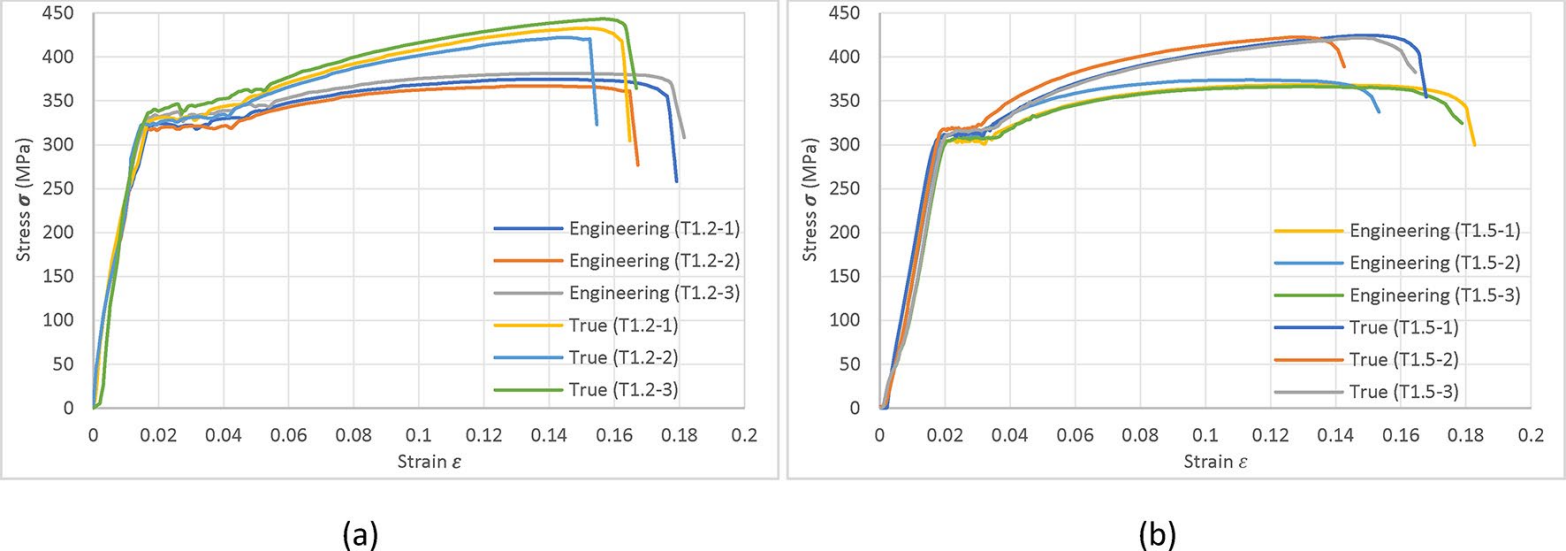
Stress (σ) – Strain (ε) curves.

For the 1.2 mm thick columns, measured yield and ultimate strengths were 321.5 MPa and 374.1 MPa, with an elastic modulus of 216 MPa. Similarly, for the 1.5 mm thick columns, the measured values were 305.4 MPa and 369.7 MPa for yield and ultimate strengths, respectively, with an elastic modulus of 205 MPa. In this investigation, three trials are conducted for each specific thickness. Subsequently, the resultant data from each trial is averaged. However, it is important to note that the averaging process is limited to the data collected up to the First Rapture within each of the three trials, (as illustrated Fig. 2).

The Poisson’s ratio for the material was established at 0.3. The stress versus strain curve for Q235 steel, obtained from the tensile coupon tests, guided the development of the corresponding true stress versus strain curve applied in the finite element models.

In the pursuit of a solver capable of providing accurate capacities and deformations aligned with experimental results, the dynamic-implicit solver in ABAQUS was selected for this study, employing a quasi-static application type^54^. To ensure convergence for both sheathed and unsheathed specimens, an equilibrium adjustment coefficient of 0.005 times the strain energy, following Rasmussen’s proposal^55^, was implemented. Additionally, in adherence to Schafer’s recommendation^56^, the displacement load was set at approximately 1.5 times the ultimate displacement, with initial and maximum loading steps established at 0.01 and 0.02, respectively. This comprehensive approach aimed to enhance calculation accuracy in the numerical simulations.

**Table 1:** Comparison of finite element analysis (FEA) outcomes with AISI and AS/NZS standards in Relation to Test results^26^ for Double-C back-to-back Built-Up columns and single columns.

Specimen	Py	*P*_EXP_	*P*_FEA_	*P*_DSM_	*P*_FEA_ / *P*_EXP_	*P*_DSM_ / *P*_EXP_	Mode
	kN						
LC3-90-A1	158.5	42.6	46.18	42.47	1.08	1.00	G
LC3-90-A2	156.7	39.9	43.56	40.44	1.09	1.01	G
LC3-90-A3	156.7	41.2	43.57	40.15	1.06	0.97	G
SC3-90-A1	157.6	127.7	127.67	128.46	1.00	1.01	L
SC3-90-A2	158.8	132.8	134.77	129.42	1.01	0.97	L
SC3-90-A3	161.4	131.6	129.02	131.65	0.98	1.00	L
LC3-140-A1	230.8	49.2	49.19	46.09	1.00	0.94	G
LC3-140-A2	232.3	46.9	51.52	48.06	1.10	1.02	G
LC3–140-A3	234.7	50.2	54.99	52.49	1.10	1.05	G
SC1-90-A1	79.8	64.3	64.46	55.07	1.00	0.86	L
SC1-90-A3	79.8	60.8	63.44	55.12	1.04	0.91	L
MC1-140-A1	123.6	46.3	44.57	41.61	0.96	0.90	LG
MC1-140-A2	122.3	41.8	44.08	41.38	1.05	0.99	LG
MC1-140-A3	122	42.5	43.12	40.45	1.01	0.95	LG
Mean					1.036	0.970	
S					0.045	0.054	

### Results of validated models

In conducting this study, fourteen experimental laboratory tests were conducted^26^. A comprehensive comparative analysis was performed using various analytical methods, namely AISI & AS/NZS Elastic Linear analysis and Finite Element (FE) nonlinear analysis, as outlined in Table 1. A comparison of experimental results with nonlinear finite element analysis (FEA) and the American Iron and Steel Institute and Australian and New Zealand standards (AISI & AS/NZS) reveals commendable agreement, particularly in predicting the buckling behavior of the columns built-up from cold-formed double C steel channels. While the results of the finite element analysis show over estimate of approximately 3.6% with a corresponding sample standard deviation (S) of 0.045 compared to experimental tests, the AISI and AS/NZ Standards demonstrate a conservatism of about 3.0% with a corresponding sample standard deviation (S) of 0.054. The relatively small standard deviation values indicate the accuracy and reliability of the analytical methods employed.

**Fig. 3:**
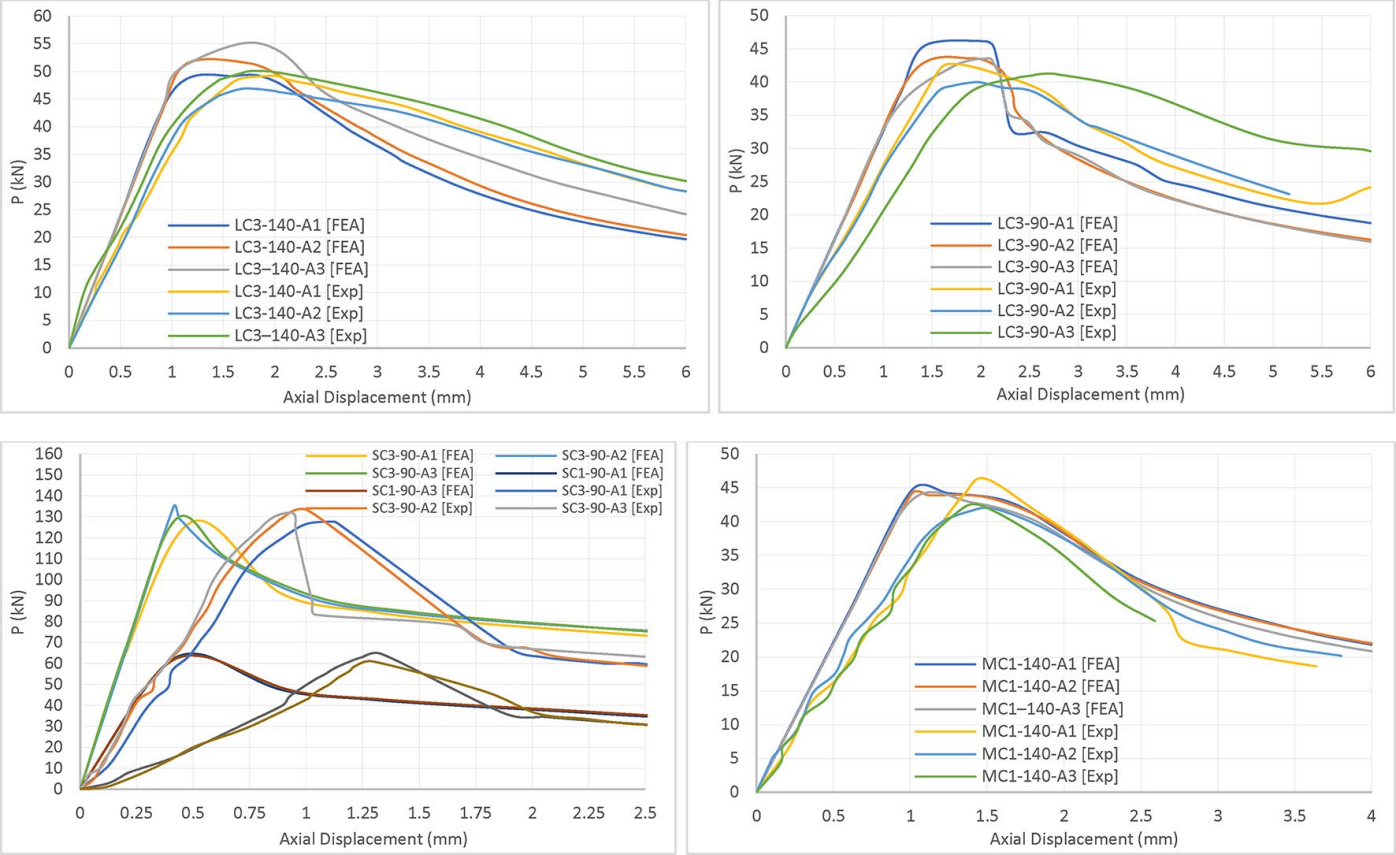
Comparing the experimental results^26^ with Finite Element Analysis (FEA) findings.

On the other hand, upon examining the axial displacement and applied load graphs for both Finite Element Method (FEM) and experimental testing (refer to Fig. 3), notable congruence in results is observed. The graphs depict a consistent trend, showcasing a Pu value being attained with a 1.5 mm axial displacement in both FEM and experimental tests. This implies a parallelism in the outcomes generated by the two methodologies.

**Fig. 4:**
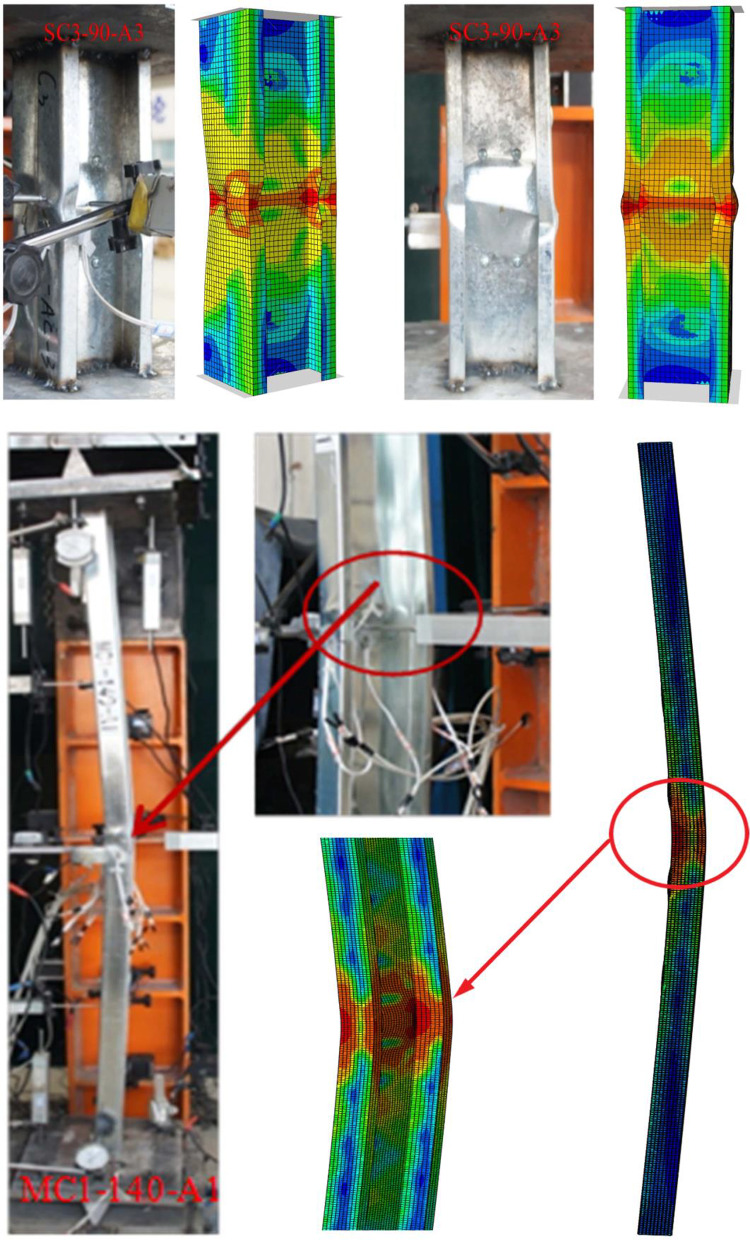
The buckling shape for both experimental^26^ and FE models.

Experimental testing and Direct Strength Method (DSM) calculations have confirmed that stub columns (SC3-90) exhibit local buckling, whereas long columns (LC3) experience global buckling. The buckling behavior observed in the ‘SC3-90-A3’ specimen was consistent in both experimental and finite element (FE) models, as depicted in Fig. 4. The visual comparison of these models demonstrates a strong correlation in buckling patterns.

It is noteworthy that the findings from both FEM and American Iron and Steel Institute (AISI) align closely with those obtained through experimental testing. Nevertheless, a subtle distinction arises, indicating that AISI tends to adopt a more conservative approach in comparison to FEM when evaluated against experimental results.

The experimental investigations, AISI linear analysis, and FE nonlinear analysis collectively contribute to a comprehensive understanding of the behavior of the tested structures. The agreement between the methods substantiates the reliability of both AISI and FEM, with nuanced variations that warrant consideration in specific analytical contexts.

## Numerical investigation

### General

In both ABAQUS and CUFSM, the material is “S355” steel, characterized by specific mechanical properties including a yield stress (F_y_) of 355 MPa, a mass density of 7.849$$\:\times\:{10}^{-9}$$$$\:\raisebox{1ex}{$\text{g}$}\!\left/\:\!\raisebox{-1ex}{${\text{m}\text{m}}^{3}$}\right.$$, a Young’s Modulus of 210,000 MPa, and a Poisson’s ratio of 0.3. Conforming to AISI S100 ^11,13^ specifications, the screw diameter is mandated to fall within the range of greater than or equal to 2.03 mm and less than or equal to 6.35 mm. Furthermore, the specifications dictate that the minimum distance between screws and edges or ends should be at least one and a half times the screw diameter, while the minimum distance between screws must be a minimum of three times the screw diameter. In the present investigation, the nominal screw diameter (d_s_) is established at 4.8 mm, with edge and end distances set at 20 mm for two lanes of screws, as illustrated in Fig. 8. When employing a single lane of screws, the edge distance equals half of the web depth (D/2).

### Loading and boundary conditions

**Fig. 5:**
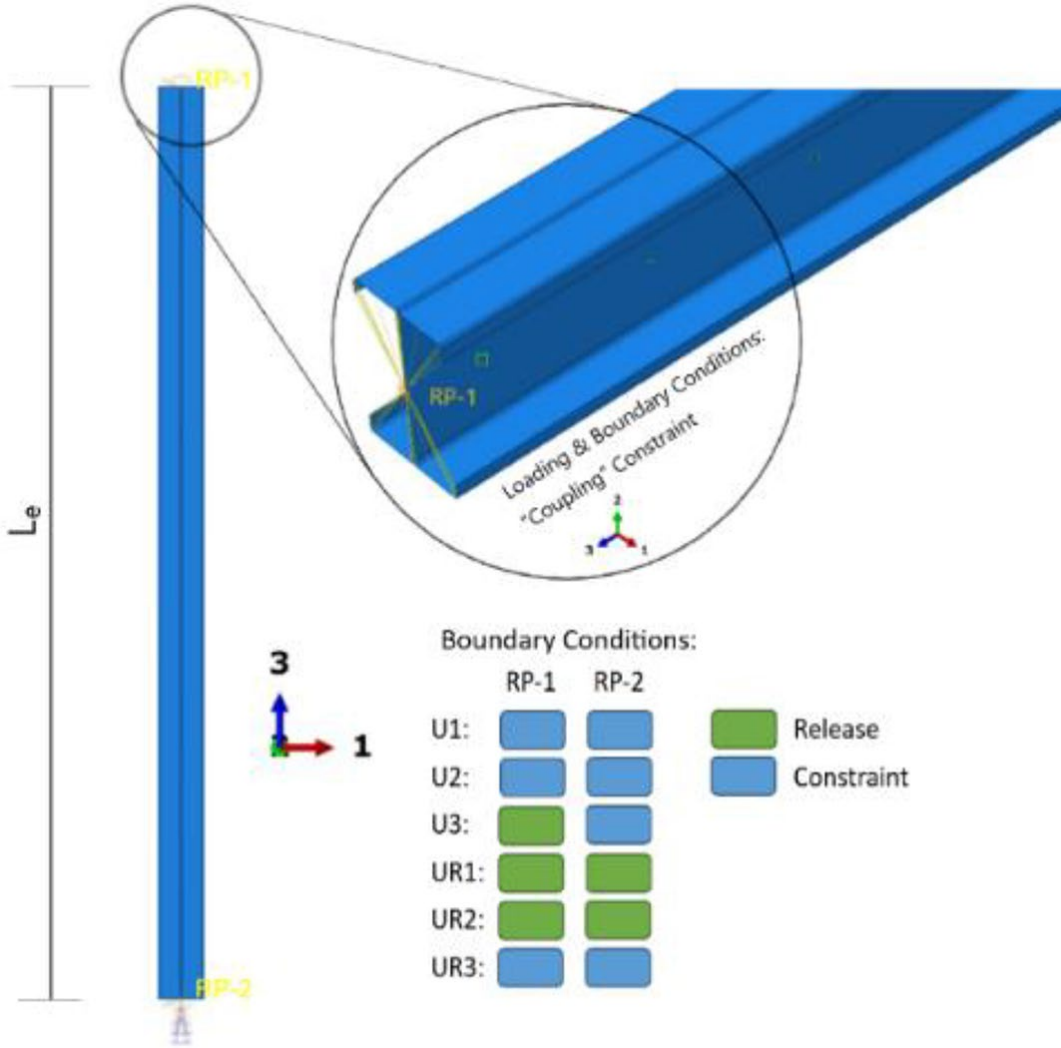
Boundary conditions of finite element model.

To establish appropriate boundary conditions, coupling constraints were initially applied to both end cross-sections of the structure. Subsequently, all degrees of freedom (displacements U1, U2, and U3, as well as rotation UR3) were restrained at the bottom reference point (RP-2). At the top reference point (RP-1), similar constraints were imposed, with the exception of the axial displacement (U3), which was left free to allow for the application of axial compression loading, as illustrated in Fig. 5. This loading was introduced through displacement control at RP-1. Both reference points were strategically positioned at the centroids of their respective members to ensure accurate representation of the structural behavior.

### Step selection in Abaqus

The finite element models were developed using Abaqus/CAE 2024 ^57^. For the buckling analysis, the “Linear Perturbation - Buckle” analysis step was utilized. A total of 200 eigenvalues were requested, with 208 vectors used per interaction, and a maximum of 100,000 iterations allowed. Linear buckling analysis (LBA) is a structural analysis technique employed to determine the critical load at which a structure or component, modeled as a linear elastic material, reaches a state of neutral stability between its unbuckled and buckled configurations.

### Mesh refinement and element type selection

**Fig. 6:**
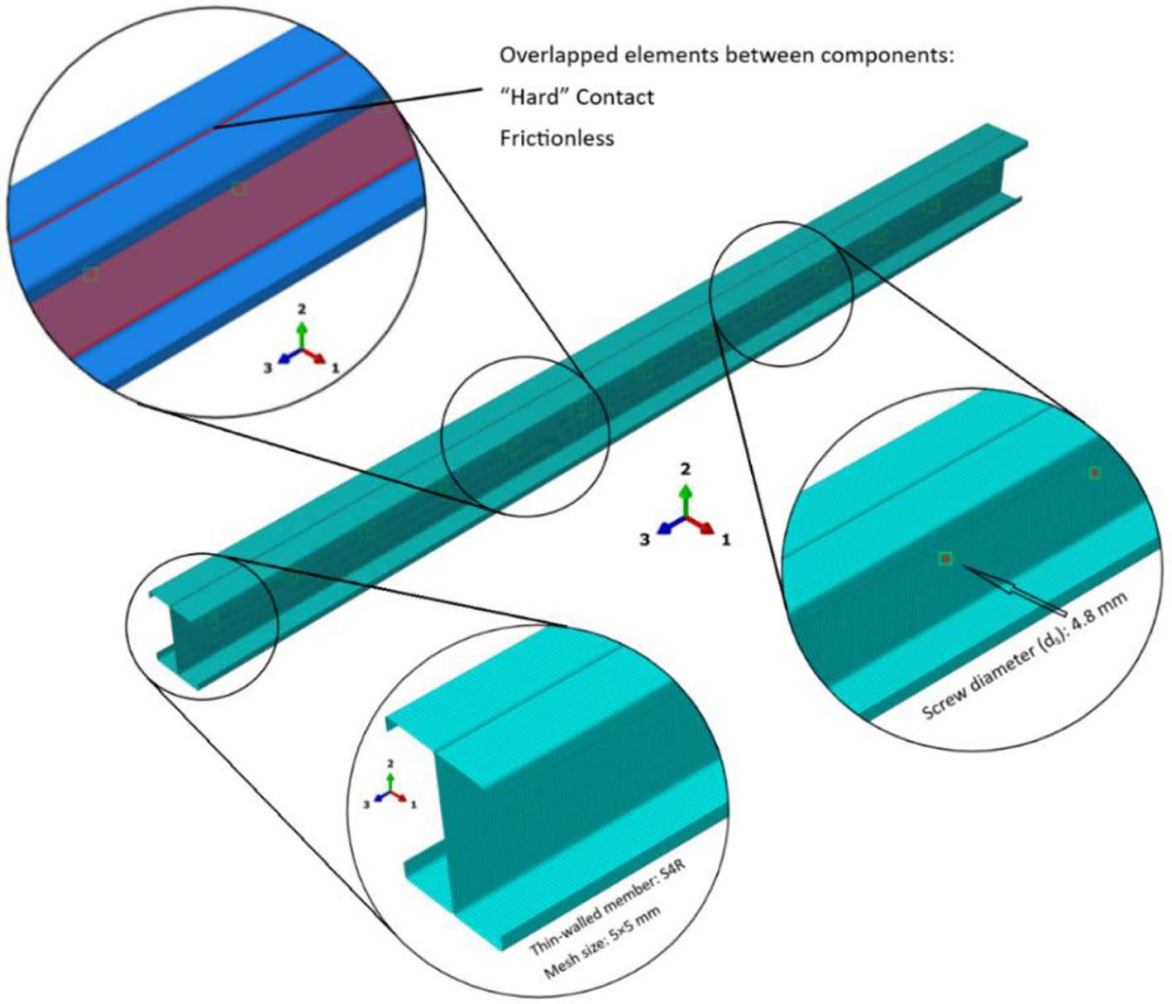
Finite element model of CFS built-up section member.

The selection of mesh size in finite element analysis (FEA) plays a crucial role in determining both the accuracy and computational efficiency of the simulation. A coarse mesh may fail to capture essential geometric details and loading conditions, thus compromising the reliability of the results. In this study, a mesh size of 5 mm by 5 mm was employed for the columns, as illustrated in Fig. 6. When the section thickness is less than one-tenth of the element size, the use of shell elements is recommended^53^ to ensure accurate representation of the structural behavior. The S4R element^55,58^ is particularly appropriate for this application, given its computational efficiency and suitability for analyzing thin-walled structures.

### Finite element model development

**Fig. 7:**
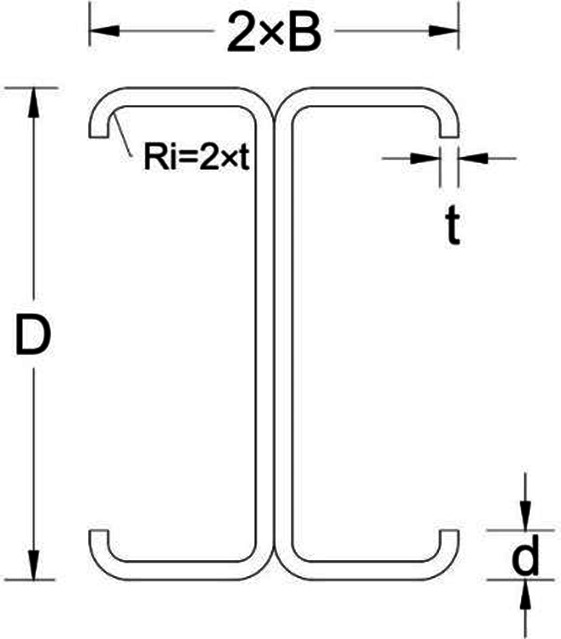
Cross-section geometry and parameters.

The cross-sections of the specimens used in this study are defined by the following equation: (2CD * B * d * t). In this equation, the coefficient “2” reflects the double back-to-back column configuration. “C” represents the C-section profile, “D” denotes the web depth, “B” indicates the flange width, “d” corresponds to the lip length, and “t” refers to the thickness of the cold-formed steel (CFS) sections. A schematic representation of the cross-sectional geometry is presented in Fig. 7.

Concerning the junctions between webs and flanges and between lips and flanges, the inside radius is set to twice the thickness of the respective cross-sections, while the outside fillet radius is established at three times the cross-section thickness of the specimen cross-sections (see Table 2).

**Table 2:** The dimensions of C-sections used as columns.

Sections	Web depth (D)	Flange width (B)	Thickness (t)	Lip length (d)	Inside radius (Ri)
	mm				
C200*75*20*2.5	200	75	2.5	20	5
C200*75*20*2	200	75	2	20	4
C200*75*20*1.5	200	75	1.5	20	3
C150*75*20*2	150	75	2	20	4
C150*65*20*2	150	65	2	20	4
C150*50*20*2	150	50	2	20	4
C100*75*20*2	100	75	2	20	4

The seven cross-sections defined in Table 2 are categorized into three groups. Initially, the first category encompasses three cross-sections characterized by identical web depth, flange width, and lip depth, but with varying thickness. Moving on to the second category, it comprises three specimens with consistent web depth, lip length, and thickness, yet differing in flange width. Finally, the third category includes three specimens with the same flange width, lip depth, and thickness, but exhibiting variations in web depth (refer to Table 2; Fig. 7).

**Fig. 8:**
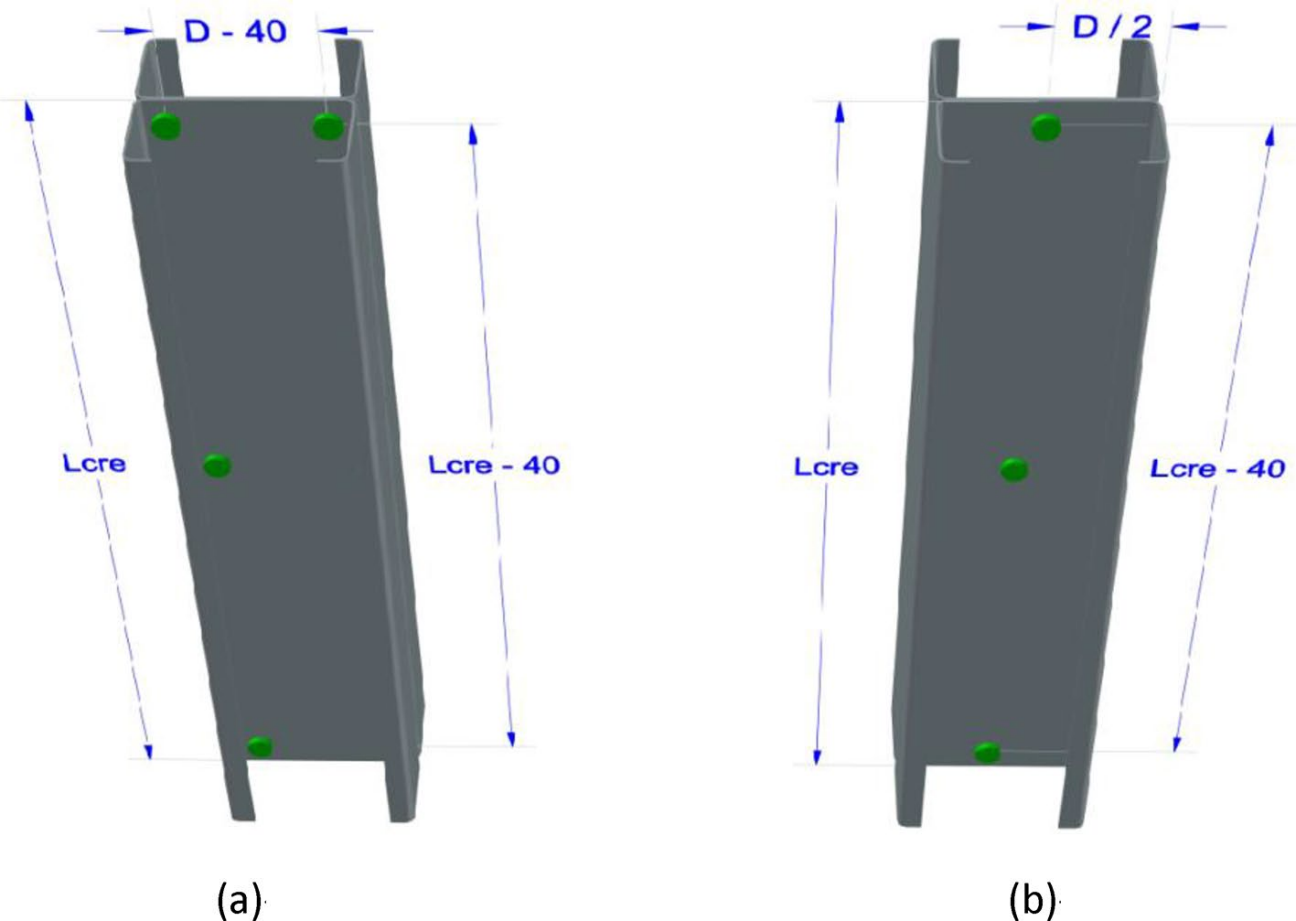
Screw Arrangement Comparison: Double-Layer vs. Single-Layer.

The seven cross-sections were utilized solely as columns, subjected exclusively to axial loading. Each column was formed by connecting two C-sections with screws. The columns were categorized into two types based on the screw arrangement: (1) columns with a single row of screws, where the back-to-back C-sections were connected along the midline of their webs, and (2) columns with two rows of screws, where the back-to-back C-sections were connected by two rows of screws, each located 20 mm from the outer edge of the flanges (see Fig. 8a and b).

The objective is to determine the optimal screw spacing that yields the maximum nominal axial strength Pn according to Eq. (13). Within each column configuration, thirteen different screw spacings are examined (see Table 3). The research incorporates a total of 175 modules with simply-supported columns created using Abaqus software (see Fig. 5). There are seven distinct back-to-back c-section columns, each comprising 25 screw spacings. These 25 spacings are categorized as follows: initially, no screws are utilized, followed by twelve different screw spacings in a single line, and finally, twelve different screw spacings across two lines.

### Determination of screw spacing

Schafer et al. introduced a software tool titled CUSFM^59^, serving as an implementation of the Effective Width Method (EWM). This software serves the purpose of identifying various half-wave lengths. This software was used to compute the half-wave lengths shown in the Table 3, where the specimens investigated in this study are comprehensively described.

**Table 3:** Specimens investigated in this paper.

	half-wave length*	(a) 2*C200*75*20*2.5	(b) 2*C200*75*20*2	(c) 2*C200*75*20*1.5	(d) 2*C150*75*20*2	(e) 2*C150*65*20*2	(f) 2*C150*50*20*2	(g) 2*C100*75*20*2
		Screw Spacing						
		mm						
S0	0 S	–	–	–	–	–	–	–
S1	L_cre_/1	2960	2960	2960	2960	2960	2960	2960
S2	L_cre_/2	1480	1480	1480	1480	1480	1480	1480
S3	L_cre_/3	987	987	987	987	987	987	987
S4	L_cre_/4	740	740	740	740	740	740	740
S5	L_crd_/1	592	592	740	592	592	493	592
S6	L_crd_/2	269	329	370	329	296	247	296
S7	L_crd_/3	185	211	247	211	197	156	197
S8	L_crd_/4	141	164	185	164	148	118	156
S9	L_crl_/1	156	156	156	118	114	114	85
S10	L_crl_/2	76	76	76	58	58	56	42
S11	L_crl_/3	51	51	51	39	38	37	28
S12	L_crl_/4	38	38	38	29	29	28	21

*The values of L_cre_, L_crd_, and L_crl_ represent half-wavelengths of elastic global, distortional and local buckling modes, respectively.

Additionally, the lengths of local buckling (L_cre_), distortional buckling (L_crd_), and global buckling (L_cre_) of each column were determined using CUFSM^59^, a software utilizing the finite strip method (FSM) to compute various types of buckling lengths. To provide a comprehensive comparison, an evaluation is conducted among all the results obtained from the Abaqus and CUFSM computations.

In illustrating spacing considerations, a straightforward model can be employed, denoted as (L_cr_/n), where L_cr_ represents the half-wavelength associated with global (L_cre_), distortional (L_crd_), and local (L_crl_) buckling phenomena, respectively. Here, n takes on values of 1, 2, 3, and 4, corresponding to each type of buckling mode. Furthermore, the notation 0 S indicates that no screws are utilized for connecting sections see Table 3.

## Results of parametric study

This study employs finite element analysis to determine the critical elastic buckling loads (local, distortional, and global) for all column configurations. Subsequently, established design codes (AISI S100 and AS/NZS 4600) are utilized, with Eqs. (2) to (12), to convert the critical elastic loads into their corresponding nominal counterparts. Finally, Eq. (13) is applied to ascertain the overall elastic nominal buckling load for each type of built-up column, where single c-sections are not connected with screws. Detailed results are presented in Table 4.

**Table 4:** Determination of the critical and nominal loads for specimens employing a zero screw (0 S).

Specimen	*P*_crl_ (0 S)	*P*_crd_ (0 S)	*P*_cre_ (0 S)	*P*_*n*_ (0 S)
	kN			
2*C200*75*20*2.5	325	490	450	294
2*C200*75*20*2	167	310	361	203
2*C200*75*20*1.5	70	168	269	124
2*C150*75*20*2	252	405	361	224
2*C150*65*20*2	241	411	253	187
2*C150*50*20*2	224	376	132	116
2*C100*75*20*2	460	460	348	244
average	248	374	310	199

When built-up back-to-back C-sections, comprising large webs and flanges, are assembled by screwing them together in a single line, the P_crl_ increases significantly, accompanied by increments in P_crd_, P_cre_, and P_n_. However, when employing two lines of screws for connection and subsequently reducing the screw spacing, P_crl_ experiences a substantial surge, while P_crd_ sees a pronounced increase, along with enhancements in P_cre_ and P_n_.

**Table 5:** The critical and nominal load outcomes for a column with dimensions (2*C200*75*20*2.5).

ID	Screw spacing	Mode	Pcrl/Pcrl (0 S)		Pcrd/Pcrd (0 S)		Pcre/Pcre (0 S)		Pn/Pn (0 S)	
	mm		%							
No. of layers			One	Two	One	Two	One	Two	One	Two
0 S	-	L	100	100	100	100	100	100	100	100
L_cre_/1	2960	L	100	100	100	100	100	100	100	100
L_cre_/2	1480	L	100	100	100	100	100	100	100	100
L_cre_/3	987	L	100	100	101	102	103	105	101	102
L_cre_/4	740	L	100	100	102	105	104	106	102	102
L_crd_/1	592	L	100	100	102	103	105	110	102	104
L_crd_/2	269	L	101	102	103	116	109	112	104	105
L_crd_/3	185	L	104	105	103	128	112	113	106	107
L_crd_/4	141	L	101	104	102	129	109	113	104	106
L_crl_/1	156	L	100	103	107	123	106	113	103	106
L_crl_/2	76	L	119	117	108	148	111	114	110	111
L_crl_/3	51	L	128	121	110	156	111	115	113	112
L_crl_/4	38	L	134	124	111	162	112	115	115	113

Changes in the thickness of these members, exemplified by (2*C200*75*20*t), lead to variations in load-carrying capacity without altering the buckling type. Similarly, modifications in screw spacing do not influence the buckling type but exert a notable impact on load-carrying capacity, as depicted in Tables 5, 6 and 7.

**Table 6:** The critical and nominal load outcomes for a column with dimensions (2*C200*75*20*2).

ID	Screw spacing	Mode	Pcrl/Pcrl (0 S)		Pcrd/Pcrd (0 S)		Pcre/Pcre (0 S)		Pn/Pn (0 S)	
	mm		%							
No. of layers			One	Two	One	Two	One	Two	One	Two
0 S	–	L	100	100	100	100	100	100	100	100
L_cre_/1	2960	L	100	100	100	100	100	100	100	100
L_cre_/2	1480	L	100	100	100	101	100	101	100	101
L_cre_/3	987	L	100	100	100	101	103	106	101	103
L_cre_/4	740	L	100	100	101	102	103	108	101	103
L_crd_/1	592	L	100	100	101	103	107	110	103	104
L_crd_/2	329	L	100	101	99	119	108	112	103	105
L_crd_/3	211	L	105	105	104	126	110	114	105	107
L_crd_/4	164	L	101	103	100	130	111	114	104	106
L_crl_/1	156	L	100	103	102	129	111	115	104	106
L_crl_/2	76	L	121	118	107	147	111	116	111	112
L_crl_/3	51	L	131	123	108	158	112	116	114	114
L_crl_/4	38	L	137	126	109	165	112	116	116	115

For instance, when considering (2*C200*75*20*1.5) with zero screws, the Nominal Axial Strength is measured at 124 kN. However, augmenting the thickness of the built-up section by 33.33% and 66.67% correspondingly increases the Nominal Axial Strength by 63.71% and 137.1%, respectively, as outlined in Table 4.

**Table 7:** The critical and nominal load outcomes for a column with dimensions (2*C200*75*20*1.5).

ID	Screw spacing	Mode	Pcrl/Pcrl (0 S)		Pcrd/Pcrd (0 S)		Pcre/Pcre (0 S)		Pn/Pn (0 S)	
	mm		%							
No. of Layers			One	Two	One	Two	One	Two	One	Two
0 S	–	L	100	100	100	100	100	100	100	100
L_cre_/1	2960	L	100	100	100	100	100	100	100	100
L_cre_/2	1480	L	100	100	100	100	100	100	100	100
L_cre_/3	987	L	100	100	100	101	101	105	100	102
L_cre_/4	740	L	100	100	101	103	101	108	100	103
L_crd_/1	740	L	100	100	101	103	106	108	102	103
L_crd_/2	370	L	102	102	102	109	109	112	104	105
L_crd_/3	247	L	103	103	103	109	108	114	104	106
L_crd_/4	185	L	104	105	104	124	101	114	102	107
L_crl_/1	156	L	100	103	105	130	109	115	103	106
L_crl_/2	76	L	122	117	105	148	110	117	111	112
L_crl_/3	51	L	131	122	107	159	110	118	114	114
L_crl_/4	38	L	137	125	107	167	111	118	116	115

When built-up back-to-back c-Sect. (2*C150*B*20*2) which is screwed together, there is a discernible impact on structural behavior. Initially, when it is screwed together with one line of screws, then by reducing screw spacing, P_crl_ increases significantly along with notable increases in P_crd_, P_cre_, and P_n_. Moreover, upon employing two lines of screws to connect C-sections, reducing the screw spacing exhibits a profound effect: it substantially elevates P_crl_, markedly augments P_crd_, and boosts P_cre_ and P_n_. These alterations in screw configurations underscore the critical role of fastening mechanisms in load distribution and structural integrity.

**Table 8:** The critical and nominal load outcomes for a column with dimensions (2*C150*75*20*2).

ID	Screw spacing	Mode	Pcrl/Pcrl (0 S)		Pcrd/Pcrd (0 S)		Pcre/Pcre (0 S)		Pn/Pn (0 S)	
	mm		%							
No. of layers			One	Two	One	Two	One	Two	One	Two
0 S	–	L	100	100	100	100	100	100	100	100
L_cre_/1	2960	L	100	100	100	100	100	100	100	100
L_cre_/2	1480	L	100	100	100	100	100	100	100	100
L_cre_/3	987	L	100	100	100	101	101	106	100	102
L_cre_/4	740	L	100	100	100	101	108	109	103	103
L_crd_/1	592	L	100	100	100	104	106	111	102	104
L_crd_/2	329	L	100	102	101	102	111	114	104	105
L_crd_/3	211	L	101	101	101	109	112	115	104	105
L_crd_/4	164	L	105	107	104	124	114	116	106	107
L_crl_/1	118	L	100	106	104	111	107	115	102	107
L_crl_/2	58	L	123	127	105	141	115	117	112	114
L_crl_/3	39	L	133	136	105	147	115	117	115	117
L_crl_/4	29	L	138	141	106	150	115	117	116	118

Additionally, changes in the flange width of these members (2*C150*B*20*2) provoke notable variations in load carrying capacity and buckling behavior. Likewise, modifications in screw spacing exert a tangible influence on buckling characteristics and load-carrying capacity. This interplay of factors is elucidated in Tables 8, 9 and 10, providing a comprehensive understanding of how design parameters affect structural performance.

**Table 9:** The critical and nominal load outcomes for a column with dimensions (2*C150*65*20*2).

ID	Screw spacing	Mode	Pcrl/Pcrl (0 S)		Pcrd/Pcrd (0 S)		Pcre/Pcre (0 S)		Pn/Pn (0 S)	
	mm		%							
No. of layers			One	Two	One	Two	One	Two	One	Two
0 S	–	L	100	100	100	100	100	100	100	100
L_cre_/1	2960	L	100	100	100	100	100	100	100	100
L_cre_/2	1480	L	100	100	100	101	100	100	100	100
L_cre_/3	987	L	100	100	100	102	102	106	101	103
L_cre_/4	740	L	100	100	100	102	103	108	101	104
L_crd_/1	592	L	100	100	101	103	105	111	102	105
L_crd_/2	296	L	102	103	102	116	110	113	105	107
L_crd_/3	197	L	101	103	102	122	114	115	106	107
L_crd_/4	148	L	106	108	106	129	109	114	106	108
L_crl_/1	114	L	100	106	105	111	112	115	105	109
L_crl_/2	58	F	123	127	107	149	114	116	113	115
L_crl_/3	38	F	133	135	107	157	114	116	116	118
L_crl_/4	29	F	139	140	108	160	114	116	118	119

Transitioning to the specific case of (2*C150*50*20*2), the absence of screws yields a Nominal Axial Strength of 116 kN. However, as the flange width of this built-up section increases by 30% and 50% respectively, the Nominal Axial Strength experiences substantial increments of 61.21% and 93.1% correspondingly. For detailed insights, refer to Table 4, which highlights the direct correlation between flange width adjustments and axial strength enhancement.

**Table 10:** The critical and nominal load outcomes for a column with dimensions (2*C150*50*20*2).

ID	Screw spacing	Mode	Pcrl/Pcrl (0 S)		Pcrd/Pcrd (0 S)		Pcre/Pcre (0 S)		Pn/Pn (0 S)	
	mm		%							
No. of layers			One	Two	One	Two	One	Two	One	Two
0 S	–	F	100	100	100	100	100	100	100	100
L_cre_/1	2960	F	100	100	100	100	100	100	100	100
L_cre_/2	1480	F	100	100	99	100	100	100	100	100
L_cre_/3	987	F	100	100	99	100	104	106	104	106
L_cre_/4	740	F	100	101	99	101	106	108	106	108
L_crd_/1	493	F	100	101	100	103	108	110	108	110
L_crd_/2	247	F	100	101	102	128	110	112	110	112
L_crd_/3	156	F	106	107	107	131	111	113	111	113
L_crd_/4	118	F	100	106	105	140	112	113	112	113
L_crl_/1	114	F	100	106	106	114	112	113	112	113
L_crl_/2	56	F	124	126	109	162	112	114	112	114
L_crl_/3	37	F	135	134	111	173	113	114	113	114
L_crl_/4	28	F	140	138	111	178	113	114	113	114

When built-up back-to-back c-Sect. (2*CD*75*20*2) are screwed together, the arrangement significantly impacts the structural integrity. Initially, when connected with a single line of screws, a reduction in screw spacing results in a notable increase in P_crl_, thereby affecting P_crd_, P_cre_, and P_n_. Moreover, employing two lines of screws for connection intensifies this effect, particularly augmenting P_crl_ and P_crd_, followed by subsequent increases in P_cre_ and P_n_.

**Table 11:** The critical and nominal load outcomes for a column with dimensions (2*C100*75*20*2).

ID	Screw spacing	Mode	Pcrl/Pcrl (0 S)		Pcrd/Pcrd (0 S)		Pcre/Pcre (0 S)		Pn/Pn (0 S)	
	mm		%							
No. of layers			One	Two	One	Two	one	Two	One	Two
0 S	–	T	100	100	100	100	100	100	100	100
L_cre_/1	2960	T	100	100	100	100	100	100	100	100
L_cre_/2	1480	F	100	100	100	100	100	100	100	100
L_cre_/3	987	F	101	100	100	100	107	109	103	104
L_cre_/4	740	F	101	101	100	100	110	113	104	105
L_crd_/1	592	F	100	101	100	100	111	115	105	106
L_crd_/2	296	F	101	102	100	102	115	119	106	108
L_crd_/3	197	F	101	103	100	100	117	120	107	108
L_crd_/4	156	F	100	101	100	103	117	120	107	108
L_crl_/1	85	F	100	115	103	116	120	121	108	109
L_crl_/2	42	F	121	142	104	125	121	122	108	109
L_crl_/3	28	F	130	155	104	127	121	122	108	109
L_crl_/4	21	F	133	161	104	128	121	122	108	109

Alterations in the web depth of these members (2*CD*75*20*2) invariably alter their load-carrying capacity. Furthermore, adjustments in screw spacing influence the buckling behavior, especially noticeable when utilizing smaller screw spacings as exemplified in (2*C100*75*20*2). These variations are elucidated in detail within Tables 6, 8 and 11.

In scenarios where (2*CD*75*20*2) is employed and the screw count reaches zero, the Nominal Axial Strength amounts to 244 kN. However, as the web depth of the built-up section increases by 50% and 100%, the Nominal Axial Strength experiences a respective decrease of 8.2% and 16.8%, as outlined in Table 4.

**Table 12:** Average outcomes of all columns for both critical and nominal loads.

ID	Screw spacing	Pcrl/Pcrl (0 S)		Pcrd/Pcrd (0 S)		Pcre/Pcre (0 S)		Pn/Pn (0 S)	
	mm	%							
No. of layers		One	Two	One	Two	One	Two	One	Two
0 S	–	100	100	100	100	100	100	100	100
L_cre_/1	2960	100	100	100	100	100	100	100	100
L_cre_/2	1480	100	100	100	100	100	100	100	100
L_cre_/3	987	100	100	100	101	103	106	102	103
L_cre_/4	740	100	100	101	102	105	109	102	104
L_crd_/1	599	100	100	101	103	107	111	103	105
L_crd_/2	305	101	102	101	113	110	113	105	107
L_crd_/3	201	103	104	103	118	112	115	106	108
L_crd_/4	154	102	105	103	125	110	115	106	108
L_crl_/1	128	100	106	105	119	111	115	105	108
L_crl_/2	63	122	125	107	146	113	117	111	112
L_crl_/3	42	132	132	108	154	114	117	113	114
L_crl_/4	32	137	136	108	159	114	117	115	115

In terms of results, a notable enhancement in nominal axial strength (P_n_) is observed. Specifically, for one lane of screws, the increase is 15%, 13%, 11%, 5%, 6%, 6%, and 5% for the corresponding screw spacings mentioned earlier. Conversely, for the configuration with two lanes of screws, P_n_ experiences increases of 15%, 14%, 12%, 8%, 8%, 8%, and 7%, respectively. See Table 12.

For economic reasons, it is advisable to avoid the utilization of L_crl_/4 and L_crl_/3 screw spacings. The rationale behind this recommendation is that these configurations necessitate a substantial number of screws, resulting in closely spaced screws. Consequently, steering clear of these two spacing types is deemed optimal.

As depicted in Fig. 8b, employing a single lane of screws along the centerline of the webs can significantly enhance the critical elastic local buckling load (P_crl_). This enhancement is particularly noteworthy when the spacing is equal to L_crl_/n, where n takes values of 1, 2, 3, and 4. With each incremental value of n, there is a notable surge in P_crl_, accompanied by a marginal increase in P_n_.

Referencing in Fig. 8a, an alternative approach involves employing two lanes of screws, leading to an improvement in the critical elastic distortional buckling strength (P_crd_). This improvement is especially pronounced when the spacing equals L_crl_/n, with n ranging from 1 to 4. Notably, as n increases, there is a substantial rise in (P_crd_) and a gradual uptick in (P_n_).

For economic considerations, it is recommended to opt for a single lane of screws and adopt a screw spacing equal to S10 or L_crl_/2. This configuration results in significant increases across critical elastic local buckling load (P_crl_), critical elastic distortional buckling strength (P_crd_), critical elastic distortional-torsional buckling strength (P_cre_), and P_n_. Specifically, there is a remarkable increase of 22%, 7%, 13%, and 11%, respectively.

Screw spacing, denoted as ‘Si,’ is characterized by the interval when ‘i’ ranges from 0 to 12. Additionally, L_cr_/n is defined for ‘n’ values spanning from 1 to 4.

**Fig. 9:**
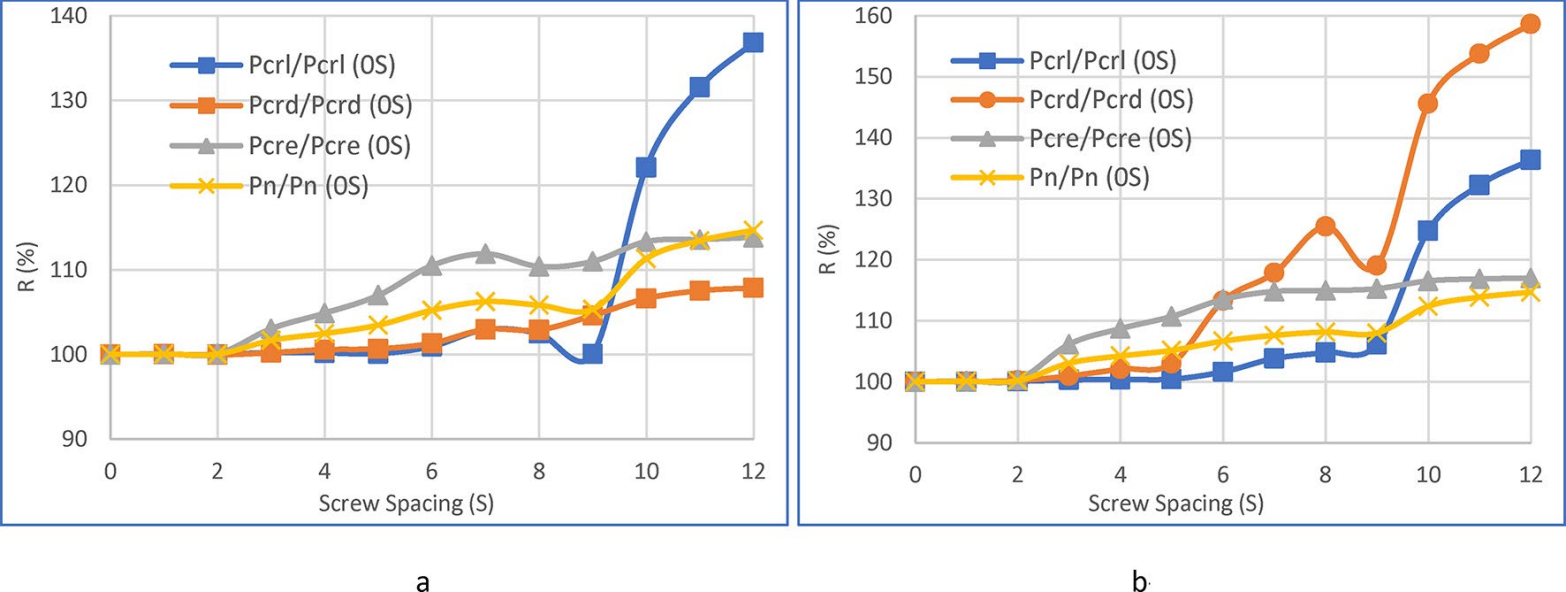
Examining the correlation between column load and screw spacing, considering both critical and nominal loads.

Referring to Fig. 9a and b, when the value is 0, it corresponds to ‘S0,’ indicating that no screws are utilized. The subsequent values, 1 through 4 on the graph, represent ‘S1,’ ‘S2,’ ‘S3,’ and ‘S4,’ corresponding to L_cre_/1, L_cre_/2, L_cre_/3, and L_cre_/4, respectively.

Similarly, values 5 through 8 on the graph, namely 5, 6, 7, and 8, correspond to ‘S5,’ ‘S6,’ ‘S7,’ and ‘S8,’ aligning with L_crd_/1, L_crd_/2, L_crd_/3, and L_crd_/4, respectively. Furthermore, values 9 through 12 on the graph, specifically 9, 10, 11, and 12, correspond to ‘S9,’ ‘S10,’ ‘S11,’ and ‘S12,’ representing L_crl_/1, L_crl_/2, L_crl_/3, and L_crl_/4, respectively.

## Conclusion

This study conducted a comprehensive finite element analysis (FEA) of 175 models to investigate the axial behavior of back-to-back cold-formed steel (CFS) channel section columns connected with fasteners. The analysis focused on local, distortional, and global buckling modes, examining seven unique channel cross-sections and twelve different screw connection configurations. The FE model’s accuracy in predicting axial load capacities and failure modes was validated through comparison with experimental test results. Additionally, the consistency between FEA, direct strength method (DSM) results, and experimental findings further confirmed the model’s reliability. Specifically, the average ratios of nonlinear FEA and DSM capacities to experimental axial load capacity were found to be 1.04 and 0.97, respectively.

Following the establishment of the credibility of the finite element models, a parametric study was conducted to explore the impact of screw spacing on various critical buckling loads and the nominal axial strength of back-to-back built-up cold-formed steel lipped channel sections. The analysis revealed that employing a small screw spacing along the centerline of the webs in a single lane significantly enhances the critical elastic local buckling load. In contrast, utilizing a small screw spacing along two lines notably improves the critical elastic distortional buckling strength. Specifically, the critical elastic local, distortional, and global column buckling loads, along with the nominal axial strength, were enhanced by 22%, 7%, 13%, and 11%, respectively, when the screw spacing along the webs’ centerline was set to half the local buckling half-wavelength. Additionally, with double-lane fasteners spaced at the same distance, these improvements were even more pronounced, achieving enhancements of 25%, 46%, 17%, and 12%, respectively.

For practical and economic considerations, it is recommended to use single-lane fasteners with a spacing equivalent to half the local buckling half-wavelength.

### Recommendation

It is recommended that future researchers and readers investigate the impact of screw spacing on the performance of built-up closed sections, as well as its effect on assemblies consisting of three or four C-sections.

## Electronic supplementary material

Below is the link to the electronic supplementary material.

Supplementary Material 1

## Data Availability

All data generated or analysed during this study are included in this published article and its supplementary information files.
